# A corpus approach to orthographic chunking: near-naive word separation in Swiss German text messages

**DOI:** 10.1515/cllt-2024-0049

**Published:** 2025-03-14

**Authors:** Erika Just, Paul Widmer

**Affiliations:** Independent Researcher, Zurich, Switzerland; University of Zurich, Zurich, Switzerland

**Keywords:** orthography, wordhood, corpus analysis, nonstandard variety

## Abstract

A lot of importance is indirectly attributed to the orthographic word: it constitutes the basis of any task that is preceded by tokenization and presents material for stimuli in psycholinguistic experiments. But in many writing traditions, the orthographic word is representative of isolated entries in the lexicon and largely ignores phonological processes of production. This study examines near-naive word separation in Swiss German using a corpus of text messages, revealing distinct patterns of orthographic segmentation driven by phonological processes such as assimilation and epenthesis. Compared to Standard German, Swiss German exhibits fewer orthographic words, suggesting heightened representation of prosodic dependencies in writing. Writers prioritize phonology over syntax when deviating from standard German space insertion conventions. These findings increase doubts about the meaningfulness of orthographic representation for word-based comparative linguistic research and highlight the importance of integrating phonological information into natural language processing models.

## 1 Introduction

It has gone down in most lines of linguistic research by now that even in widely used and well-established writing systems, the orthographic word need not correspond to any word-like units on different linguistic levels (Dixon and Aikhenvald 2002: 8; Gil 2020: 12; Haspelmath 2011). This is true for morphosyntactic as well as phonological words, which do not necessarily overlap. What is more, even within these different levels of linguistic analysis, we find a lot of internal variation, which means that there can be different types of phonological and morphosyntactic words within one single system (e.g., Bickel and Zúñiga 2017; Sampson 2016).

Although it is hard to identify basic building blocks on the morphosyntactic level, and basically futile to represent them orthographically in a uniform manner, it is in fact the orthographic word, which is often considered to mirror a basic structural unit. It is given a central role, for instance, in lexical typology, historical linguistics, corpus linguistics, L1 and L2 research, or psycholinguistics, where it is a popular object of study when it comes to language storage and processing. Tasks involving tokenization, such as NLP, heavily rely on it in languages that employ word separation in their script. Spaces and other separators are thus attributed a lot of importance.

The nature of our orthographic word is essentially syntactic (see e.g., Trask 2004: 4), so one word usually corresponds to one part of speech. But it is also semantically characterized: many definitions of “word” revolve around it being a conceptually coherent unit (see discussion in Dixon and Aikhenvald 2002: 5–11). This conforms with our intuition that there are parts of speech – particularly nouns and verbs (Wray 2014) – which constitute more prototypical words as compared to others, as they are more easily defined semantically than pronouns or determiners.

Functional units, on the other hand, can cause more uncertainty when it comes to word delineation in nonstandardized writing: Investigations of writings produced by semi-literates suggest that these are most affected by conflicting – phonological versus syntactic – drivers for space insertion (Wray 2014: 733). This particularly affects function items coming in the form of clitics and particles: Native speakers’ perception of words in previously unwritten languages seem to differ most notably with regard to the categorization of clitics (e.g., Busch and Fleischer 2015; Peterson 2011), which is not surprising given that clitics often differ in terms of syntactic and phonological dependency.

The orthographic word as found today in languages like English or German is the result of traditions and, in part, authoritative decisions. Until the Middle Ages, scriptio continua was prevalent in Europe.1Although word division by means of spacing or other dividers can be found already in writings from antiquity, this was hardly ever employed in a consistent manner (Waal 2020). The common view is that space insertion originated in Ireland in the seventh century, and spread from there (Saenger 1997). It has been argued that the emergence of spaces was due to the scribes’ unfamiliarity with Latin (Clanchy 2012: 132) and served as some kind of aeration, akin to taking a breath, facilitating the processing of the written word, especially in silent reading (Saenger 1997). Since then, these visual units have been conventionalized and entrenched over centuries.

That visual word separation is preferred over continuous script might be related to the fact that inserting spaces (and other word dividers dissimilar to orthographic symbols) facilitates reading, even in readers of scripts that can do without. For instance, Kohsom and Gobet (1997) found that adding spaces to Thai script makes reading more efficient. The same seems to hold for inserting spaces in Chinese script (Bai et al. 2008), and also for dividing up three-lexeme-compounds in German (Inhoff et al. 2000). Conversely, omitting spaces from scripts that usually employ them slows down reading in most skilled readers (Morris et al. 1990; Pollatsek and Rayner 1982; Rayner et al. 1998, Rayner and Pollatsek 1996; Spragins et al. 1976).

Given the importance of chunking for processing on the one hand, and the conventionalized character of the orthographic word on the other, it would be interesting to see what chunks emerge between spaces naturally, and what kind of unit – phonological, morphosyntactic, or lexical – they would have an inclination toward. This is especially interesting in the light of the often discussed syntax–phonology interface when it comes to clitic placement (e.g., Klavans 1995).

In a little experiment with speakers of a previously unwritten language (Kharia, Munda), Peterson (2011) finds that – although not all speakers were in unison with their analyses – where morphosyntactic and phonological boundaries did not coincide, priority was given to phonology to mark word separation, which in some cases led to the splitting up of morphemes. Similarly, Crellin (2022) finds for inscriptions in Ugarit alphabetic cuneiform, linear alphabetic Northwest Semitic, and some Ancient Greek inscriptions from the first half of the 1st millennium BCE that word division is primarily induced by prosody and phonology, rather than by morphosyntactic considerations. Needless to say, also in these inscriptions, there were writer-specific differences.

Also Alemannic speakers (probably just like speakers of any nonstandardized variety) have been known to show a lot of variation in their writing practices. Even within individual writers, there can be inconsistencies, not only in terms of orthographic sound representation but also regarding orthographic word boundaries. Nübling (1992: 213–214) provides an example from a Bernese German novel where within the same prepositional phrase, the article preceding the noun can be either chunked onto the preceding preposition (*it schueu* into:DEF school), onto the following noun (*i tschueu* into DEF:school), or the whole phrase can even comprise a single chunk (*itschueu* into:DEF:school). Such varied behavior of articles within PPs has also been reported for early writings before standardization, for instance for Old High German and Old Low German (Busch and Fleischer 2015) or Old Saxon, Old Irish and Latin (Bronner et al. 2018). Section 2.2 will go into detail about the elements that are particularly prone to varied behavior in terms of orthographic chunking.

According to Nübling (1992), there are other factors apart from prosodic dependency and conceptual coherence that can influence the degree of fusion, and thus potentially orthographic word separation, around functional items in nonstandard varieties of German. For one thing, she mentions a high bigram frequency as facilitating the merger of two words, where a bigram refers to a pair of consecutive words or characters in a given text (Nübling 1992: 57, 96, 121). She does not elaborate on the underlying processes, but it might have to do with principles of economy (high frequency leads to shortening), as well as stronger cohesion between frequently adjacent items (e.g., Bybee 2003).2High frequency has also been found to have influenced solid writing of compounds in Early New High German, see Dücker (2018). Another possible factor are the mechanisms underlying the general suffixing preference (Nübling 1992: 240, 278). Let it be supposed that language processing and production mirror in visual word separation, one would expect chunks like *it schueu* to be preferred over chunks like *i tschueu*, in compliance with this preference.

The asymmetry in terms of affixation is well-known cross-linguistically. Postposed grammatical markers are much more likely to become affixes than preposed grammatical markers. Different explanations have been proposed in the literature: Research on word recognition and production suggests that word onsets are particularly salient and important for both comprehension and production. The beginning portion of a word seems to be the most effective cue for successful recognition, and distortions or mispronunciations at the onset of a word have a greater impact on recognition than those at the end (e.g., Hawkins and Cutler 1988).

Himmelmann (2014) states that this asymmetry is related to other asymmetries between preposed and postposed function words with regard to their prosodic chunking: there are more commonly prosodic breaks between preposed grammatical elements and lexical hosts than between lexical hosts and postposed grammatical elements. This reasoning is in line with the fact that there is also an asymmetry with regard to clitic placement: ditropic clitics, i.e., enclitics attaching to a phonological host that precedes their lexical host (e.g., *we’ll see*), albeit not particularly common, are found across different language families of the world, including Indo-European languages (Himmelmann 2014). The opposite, in contrast, where a functional postposed element is proclitic to the following element, is not attested (Cysouw 2005).

However, assuming a general bias toward suffixation fails to explain that the degree of the suffixing preference varies based on grammatical categories (Cysouw 2009). For instance, whereas morphological case and nominal number are in the majority of languages realized via suffixation (Dryer 2013a, 2013c), in verbal person marking, prefixing is just as common as suffixing (Cysouw 2003).3The reason for this does probably not lie in possible grammaticalization paths alone. Taking the category of negation, for example, Dryer (2013b) states that although preverbal negation particles are by far more common than postverbal negation particles, when it comes to affixes with the same function, we find roughly the same number of negation prefixes and suffixes across languages. This observation is in accordance with the fact that the suffixing preference is cross-linguistically stronger in nouns than in verbs. The question of whether the discrepancy between nouns and verbs is an epiphenomenon of the preference for suffixation in particular grammatical categories (possibly due to scope), or whether it is the other way round and the universal bias toward suffixation is stronger for nouns than for verbs, which in turn leads to an incline of particular categories to be suffixed, remains to be answered.

According to Seifart et al. (2018), the discrepancy between nouns and verbs is related to the fact that speech slows down more before nouns than before verbs. Also nouns themselves are overall pronounced with greater duration than verbs (see Lohmann 2020 for a recent discussion of the reasons for prosodic differences between nouns and verbs.). Seifart et al. (2018: 5723) assume that when speech is slower and contains more pauses before nouns, it is less likely for independent function words to become reduced and fuse in this position. This would account for the fewer instances of function words attached as prefixes to nouns compared to verbs.

So there are numerous factors that potentially influence visual word separation, some of which have just been outlined. We find linguistic aspects like syntagmatic relationship, part of speech class, lexical cohesion, phonological dependency, and prosodic coherence. But there are also other factors like frequency effects and cognitive mechanisms facilitating processing, as well as those possibly underlying the suffixing preference. On top of that there is an additional layer of rules and conventions from a learned standard, which might interfere with what writers would do intuitively.

Given that individuals learning to write also typically acquire standardized orthographic rules, it is hard to come by data on naive writing and space insertion. However, the context of Swiss German presents an opportunity, as writers, despite having proficiency in a standard system, very often opt for nonstandardized writing. This choice allows us to observe more naturally occurring space insertion. Notably, to our knowledge, there is no precedent for a similar study delving into this specific aspect of nonstandardized German variety.

I order to explore visual word separation in Swiss German (henceforth gsw), we use a corpus of text messages. Based on what is known about nonstandardized space setting and the role of phonology and prosody, we hypothesize that there will be deviations from the standard orthographic rules. More precisely, due to the high number of prosodically dependent forms (which will be more elaborated on in Section 2.2), we expect overall fewer orthographic words (i.e., larger chunks) as compared to the standard. We also expect frequency effects, with common bigrams being supposedly written as a unit more often, as well as function words with a particularly high token frequency to be more likely chunked onto a preceding or following lexical item than those with a lower token frequency. It would also be interesting to see an effect of the suffixing preference in that word delineation is preferred immediately before lexical elements, and whether there is a difference between nouns and verbs, with everything else being equal.

It is essential to note at this point that a quantitative analysis considering individual tokens as data points faced challenges due to the high variation in spelling in the corpus resulting from dialectal differences. This variability necessitated a more nuanced approach to capture the intricacies of visual word separation in gsw.

To go about the questions above, the remainder of paper is structured as follows: Section 2 gives some background on Swiss German, its use, the corpus, and some relevant structural features mentioned in the previous literature on orthographic juncture (Section 2.2). Then, in Section 3, we will first describe some basic findings concerning the frequency of chunking certain parts of speech, followed by a more thorough description of particularly common combinations (Sections 3.2 - 3.5). Section 3.6 then discusses conjoint writing from the perspective of the suffixing preference, before we conclude the paper with a discussion in Section 4.

## 2 Swiss German

### 2.1 Language background and corpus

Swiss German (autonym Schwiizerdütsch) is not a single variety, but a conglomerate of Alemannic dialects spoken in German-speaking Switzerland. It is used on a daily basis in all areas of life, and thus registers, by most speakers, and plays an important role in the identification with regional affiliation. In school, mostly Swiss Standard German is used, which is also the standardized written form for newspapers and books. This variety is largely identical to the standard German varieties used in Germany or Austria. Most differences can be found in spelling and vocabulary, and there are a couple of minor structural differences. So people speaking a variety of gsw are usually fluent in Standard German as well.

The corpus used for this study, the Swiss SMS Corpus (Stark et al. 2009–2015), comprises chats in all four national languages of Switzerland and their different dialectal varieties. The relevant subcorpus contains 288.434 tokens from 10.674 text messages with gsw as the dominant language, collected in fall/winter 2009/2010.4The corpus of text messages has now been pooled with the newer “Whats up, Switzerland” corpus of WhatsApp messages (Stark et al. 2014–2020) and both can be accessed on https://corpora.linguistik.uzh.ch/annis/. It is annotated for various linguistic levels (lemmata, parts of speech) and contains metadata on writers for about two thirds of the data. It is hosted by and queryable through the ANNIS interface, which is an open-source, web browser-based search and visualization architecture for linguistic corpora (Krause and Zeldes 2016).

Although there is a Swiss Standard German written variety, in text messages, people often use a way of spelling that deviates from the norm, representing their dialectal variant (Dürscheid and Stark 2013).5This paper is concerned with grapheme-phone correspondences and the regularities found therein, but does not address any of the questions tackled down in the present paper. As is characteristic for sms corpora in general, writers communicate informally, using a low register and chatting about all kinds of topics, in a manner very close to spoken language (Thurlow and Poff 2013). The use of nonstandard orthography text messages is often an important means of expressing one’s identity (Sebba 2007). And as has been stated above, expressing regional identity through one’s dialect is particularly important in the Swiss Confederation. Therefore, the corpus provides a great source for spontaneous writing, without the need of adhering to standardized rules.

### 2.2 Junctures in Swiss German

As has been illustrated with the example from the Bernese novel in the introduction, visual word separation in written gsw is often not in line with the orthographical conventions that writers know from Standard German. But despite the variance within gsw, there are some lexical categories with a clear tendency to be attached to a preceding or following element. According to Nübling (1992), this pertains most notably to articles and pronouns, and often prepositional phrases. Both categories are strongly affected by phonological dependencies different from the standard variety and thus show the highest degree of divergence in terms of visual word separation. We thus expect to find a high degree of deviation from the standard orthography with these categories.

In Nübling’s analysis, these categories are in the transitional zone between syntax and inflection, and she thus classifies them as clitics. We do not wish to engage in any discussion concerning the morphological status of particular elements being clitics or not (see Haspelmath 2015 for an overview of the most important points), as the respective categorization may vary depending on the language, as well as the theoretical framework employed. Irrespective of their potential morphosyntactic categorization, articles and pronouns can display different behavior in terms of prosody or syntactic attachment from their corresponding forms in related language varieties.

Pronominal systems in dialectal variants of German often exhibit more paradigms than can be found in the standard, including phonologically reduced forms.6According to Wermke et al. (2009: 270, 871), the stressed and unstressed forms of the Standard German pronouns are formally identical, and only the 3SG.N.NOM/ACC *s* is sometimes found as a reduced bound form. Those “clitic” or “unaccented” forms are more frequently used than the full forms. Such a system has been described in detail, for instance, for West Central Bavarian (Altmann 1984), Brazilian Pomeranian (Postma 2019), or Palatinate Dialects (Green 1990). As for gsw varieties, Nübling (1992) describes a two-way distinction of full and reduced forms for Bernese, and similarly Reese (2007) for the Zurich variety. Important for our questions is the fact that usually only the reduced forms are written conjointly as part of a complex chunk. However, they don’t have to be, and there are plenty of examples in the corpus where a short form is written as a separate word, as in examples (2) and (3) below.

The two paradigms do not only differ with regard to phonological realization, but they are also functionally distinct. The different forms are used to organize information in discourse: as is cross-linguistically common, the reduced forms can be used for given and readily available information, whereas only the full forms can be used for emphasis or other focal effects. However, whereas the short forms are confined to given information, their use is not obligatory in any context, and the full forms can syntactically appear everywhere. This is exemplified in (1)–(4) from the corpus: In (1), the full form of the 2SG pronoun *du* has to be used, as it contrasts with *dini schwö* “your sister.” In example (2), the short form *d* can be used, as it is readily available information, while the time of coming over is the requested, rhematic information. Similarly, in (3), the variant *t* (attached to *wen* “when”) is used. However, as (4) shows, the scope of use of the full form overlaps with that of the short form, as this is an example where 2SG is definitely the theme, but still the full form *du* is used.7In the following, examples from the corpus will be tagged with a four to five digit number in square brackets, referring to the document name (i.e., text message) within the gsw-tagged corpus.

(1)

**Table:** 

Hej	bisch	du	das	gsi	oder	dini	schwö
hey	be.2SG	2SG.NOM	DEM	be.PTCP	or	2SG.POSS	sister
“Hey, was that you or your sister?” [11382]

(2)

**Table:** 

Säg	mer	doch	äfach	wänn	d	vebi	chunsch
tell.IMP	me	MOD	simply	when	2SG.NOM	over	come:2SG
“Just let me know when you’ll come over.” [14807]

(3)

**Table:** 

Chasch	alüte	went	chunsch
can:2SG	call.INF	when:2SG.NOM	arrive
“You can give me a call when you arrive.” [10057]

(4)

**Table:** 

hettisch	di	no	welle	träffe?	wieso	besch				
AUX.COND:2SG	2SG.ACC	still	want:INF	meet.INF	why	be.2SG				
du	so	spot	hei							
2SG.NOM	so	late	home							
cho?										
come.PTCP										
“Did you want to meet? Why did you come home so late?” [5765]										

The situation is similar for articles, especially in combination with prepositions, with which they often fuse. Already Hartmann (1980) argued to consider reduced forms of articles in spoken registers of German as forming their own paradigm for pragmatic reasons (also see Studler 2011 for gsw). The short forms are typically used to modify entities that are identifiable unambiguously, either in general or in the situational context, whereas the full forms are used for phoric reference. According to Nübling (1992: 41–42,167–184), this difference is gradual, and there are contexts where both variants would be a legitimate choice.8A very interesting case is the definite article *di* for SG.F.NOM/ACC or PL.NOM/ACC in all genders: In its reduced form, it assimilates partially or even completely to the initial sound of the following noun in most gsw varieties (cf. Nübling 1992: 201–203). Thus, we find *dWelt* “DEF:world” but *pFrou* (*dFrou) DEF:woman, *gGeiss* (*dGeiss) “DEF:goat” or even *Zit* “DEF.time.” However, before adjectives and substantival adjectives, these reductions are blocked and a syllabic allomorph *di* appears regularly, e.g., *di Gliche* (*dGliche) “DEF same.”


Thus, the differences between the paradigms cannot only be attributed to reflecting phonetic differences, and they are not in free variation. Context and co-text, common-ground-management, as well as style (Nübling 1992: 165) play a role in the choice of the reduced and thus prosodically dependent variant over a full form.

Due to the high degree of variation in the orthography (cf. Section 3.2), as well as the fact that the unaccented forms are formally often identical to the accented forms (needless to say, in writing), it is hard to find clearly contrasting examples for article use in the corpus. Also, as with pronominals, the use of an unaccented variant does not necessarily lead to conjoint writing. But if we assume that phonological reduction – and this entails not only fewer segments, but also the absence of stress in certain contexts – influences whether two items are written conjointly, one should keep in mind such pragmatic distinctions.

Presumably, different pragmatic distributions bring about different frequencies. As pronominals and articles are functional elements, they already have a very high token frequency. As pronominals in most cases refer to given, readily available information, the short forms are assumed to be more frequent than the full forms. This is confirmed by a quick frequency analysis of the test case 1SG.ACC, which displays a relatively low degree of orthographic variation in the corpus: the full form is realized as either *mich*, *mech*, or *miich*, depending on dialectal differences, and the short form is *mi*. We get 896 tokens of the short form, and only 360 tokens of the long form. The different frequency distribution also leads to the short forms forming bigrams with other items more often. Interesting to note at this point is that of the 896 short form instances of 1SG.ACC, only 76 are chunked onto a preceding item. All instances of the full form are independent orthographic words.

Additionally to the choice of paradigm, the frequency of particular types of bigrams is naturally conditioned by syntax: between prepositions and articles, for instance, nothing can intervene, their order is fixed (in gsw as well as in Standard German), whereas between an article and a noun, there can be modifiers. We would, therefore, expect the degree of fusion between prepositions and articles to be higher than that between articles and nouns. And in fact, prepositions and articles often fuse phonologically in gsw (e.g., Reese 2007: 20). Similarly, prepositions form a single stress group with a following unaccented personal pronoun, the stress falls on the preposition (Reese 2007: 25). So, although articles can also attach to a postposed noun (more on that in Section 3.3), the literature suggests that within prepositional phrases, we would find orthographic chunking of the type *it schueu* “into:DEF school” (cf. Section 2.1) more often than the type *i tschueu* “into the:school.” Here, the article (F.SG) is reduced to a single *t*, but the prediction also holds for articles which form their own syllable, for instance *em* N/M.DAT. Thus, a chunk formation like *ufem Tisch* “on:the table” should be preferred over something like *uf emTisch* “on the:table.” Addtionally, Nübling (1992: 240, 278) suggests that this might also be a manifestation of the general suffixing preference observed cross-linguistically.

## 3 Corpus study

### 3.1 Chunking frequency

**Figure 1:**
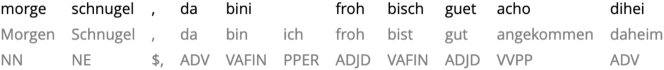
Annotation example chat [10016].

The first question, whether the degree of chunking in written gsw differs from the standard, can be answered in a quite straight-forward manner thanks to the annotation, which entails a literal word-by-word translation to Standard German (lemmata). Thus, an empty slot in the target line with a nonempty gloss corresponds to a mismatch in written word boundaries, as shown in Figure 1. The actual translation of this example into Swiss Standard German would be *Morgen Schnugel, da bin ich froh bist du gut angekommen daheim* (“Good morning sweetheart, I am happy you arrived safely at home”).

This annotation scheme does not only enable to detect mismatches in word delimitation, but it makes it also easy to look into what items combine into chunks. In this example, we find gsw *i*, which corresponds to the lemma “ich” and the part of speech personal pronoun (PPER) chunked onto the preceding auxiliary verb (VAFIN) “bin” (eng. “be.1SG”). The reverse, where we find word separation added in gsw where it would be unexpected based on the orthographic rules of the standard, are very rare and confined to cases which are prone to spelling errors also found in writers speaking Standard German. These include compound nouns like *Zeichner tagung* [10186], or particle verbs as in *di gli wieder xeh* [6036]. These cases are thus not further elaborated on.9We refer the interested reader to the endearing term “Deppenleerzeichen,” which can be translated as “fool’s space.”


Thus, the first hypothesis, that there are fewer orthographic words in gsw text messages than one would find in the standard orthography, can be easily verified: The corpus entails 287.882 glosses, corresponding to lemmata, which would be written as separate words in the standard orthography. Of these, 10.338 have an empty token (sans abbreviations), which means that there are 10.338 less orthographic word units in the text messages than we would have in a word-by-word translation into Swiss Standard German.

Due to the annotation, we can also take a look at the most frequent chunk combinations in terms of different parts of speech.10A list of abbreviations for the parts of speech can be found at the end of the paper. Small adjustments were made from the corpus export, such as merging different categories of adjectives as well as wh-words. We get 268 different types of PoS chunks, the most common of which (>20 tokens) are shown in Table 1.11To obtain this table, we removed abbreviations, some indications of time of day (like *halbi 6* which was given as “halb Uhr 6” in the annotation and would have given a false positive in terms of a chunk), as well as some items that can be considered lexicalised like *imfall*, *demfall*, or *nüm(m)(e)*. The most common chunks involve verbs (full verbs, auxiliaries, as well as modal verbs) to which personal pronouns are attached: these account for >44 %. The second most common combination are prepositions (PREP) with articles. These findings are in line with what has been stated in the literature (Section 2.2) and seem to be a reflex of the phonological dependency of pronouns and articles. Also, it shows that articles are more often chunked onto a preposed preposition than on a postposed noun. It is worth zooming in on some details in Section 3.2.

**Table 1:** Most frequent orthographic chunks in terms of parts of speech.

PoS Bigram	Frequency	Percentage	Example	Gloss
VVFIN PPER	2,217	21.7	brucheder	need:you.PL
VAFIN PPER	1,646	16.1	bini	am:I
PREP ART	1,645	16.1	abem	from:the
CONJ PPER	952	9.3	dasi	that:I
VMFIN PPER	728	7.1	chanen	can:him
ART NN	270	2.6	dwelt	the:world
ADV ADV	248	2.4	auno	even:more
WH PPER	196	0.9	woni	which:I
ADV NEG	181	1.8	nonig	not:yet
PTZU VVINF	164	1.6	zcho	to:come
ADV ADJ	123	0.6	zfrüeh	too:early
PPER PPER	88	0.9	mers	we:it
PREP NN	82	0.8	zobig	at:night
ADJ NN	71	0.7	Nöchschtmal	next:time
ADV ART	55	0.5	sone	such:a
PREP PPER	48	0.5	bisi	until:I
VVFIN PPER PPER	46	0.5	besprächemers	discuss:we:it
VAFIN ART	43	0.4	hanen	have:a
PREP NE	34	0.3	zKillwange	at:Killwangen
ART ADJ	60	0.3	echli	a:little
PPER VAFIN	31	0.3	sisch	it:is
VAFIN PPER PPER	29	0.3	händers	have:you.PL:it
PREP PIS	26	0.3	vorallem	above:all
PPER VVFIN	22	0.2	sgitt	it:is.there
VMFIN PIS	22	0.2	chame	can:you.generic
VVIMP PPER	21	0.2	sägem	tell:him

It is also remarkable that not everything we find as a written chunk that deviates from standard orthography can be attributed to the phonological reduction of one of the elements, as described in the previous literature for articles, pronouns, and prepositions. These cases include, for instance, two adverbs, an adverb and the negation particle, the infinite marker “zu” and a verb, or an adverb and an adjective (examples provided in Table 1). What these types have in common is that they form conceptual units, with a strong semantic as well as syntactic link. Therefore, there seem to be other elements besides phonology and bigram frequency, which potentially lead to chunking in written gsw. Another noteworthy observation that goes into a similar direction are formations in analogy to orthographic rules for similar structures, for instance regarding particle verbs: hits like *zmittagässe* “to eat lunch” are also quite frequent, and that they are written as a unit is likely influenced by Standard German orthographic units like *krankschreiben* “write s.o. a sick note” or *überdenken* “rethink” (derived from *krank* “sick” and *schreiben* “to write,” and *über* “over” and *denken* “think,” respectively).

In German grammar literature, verbs forming units with items like *sick* and *over* are usually labeled as particle verbs. The particles are word-forming elements in verbs that also occur elsewhere as independent words and can belong to different word classes. Most commonly one finds prepositions and adverbs, but also adjectives and nouns are possible. Some adjectival particle verbs like *krankschreiben* are partly individual cases that can be explained by the contraction of a syntactic compound, which were understood as one word. Depending on syntax, something can intervene between the individual parts, they are thus sometimes found written conjointly, sometimes apart.12For instance *Ich hoffe, dass der Arzt mich krankschreibt*. “I hope that the doctor writes me a sick note” versus *Der Arzt schreibt mich krank*. “The doctor writes me a sick note.”


Table 1 also shows that some chunked trigrams are quite common, namely those involving a finite verb and two pronominals. Checking for all cases in the corpus, including those not listed in the table, reveals that in fact all instances of chunked trigrams involve two pronominals as the second and third element, respectively. Apart from verbs, there are also conjunctions as hosts, such as *dasis* (that:I:it) or *falsidi* (if:I:you). Also, the only quadrigram in the gsw corpus *öbisdr* (if:I:it:you) consists of a conjunction and three pronominal forms. Examining the trigrams in more detail, we find that the most common third item (56 %) is the 3SG, realized as *s* or *z*. This is not surprising, given that this reduced form is phonologically dependent, as it cannot form its own syllable, and is written conjointly even in some registers of written Standard German (Wermke et al. 2009: 270, 871).

The following sections will focus individually on the most common chunks, namely prepositions and articles (3.2), articles and nouns (3.3), conjunctions and pronouns (3.4), and verbs and pronouns (3.5), and will discuss whether there is a potential aptitude for pre- versus suffixation in particular parts of speech (3.6).

### 3.2 Prepositions and articles

The combination of preposition and article is overall very frequently written as a unit, namely in 39 % of cases (1732 out of 4390).13Some demonstratives are homophonous with articles, but they cannot be phonologically reduced and are not chunked in the corpus; also, they would not show up here anyways as they are annotated differently, see https://whatsup.linguistik.uzh.ch/01_corpus/02_preprocessing/06_pos, July 19 2023. However, differences can be observed based on the particular combination of prepositions and articles. Table 2 shows the 14 bigrams with >100 occurrences in the corpus (“counts total”). Above in Sections 1 and 2.2, we talked about the potential role of bigram frequency, but the table shows that there is still a high degree of variation in terms of chunking in this subset, ranging from 1 % to 81 %.14In the whole corpus, there is one preposition + article bigram, which is always chunked, namely “at a.F,” *an einer* in the standard variety, realized as *anere* or *anäre*. It does not appear in Table 2 because its overall frequency is very low, there are only 15 occurrences.


**Table 2:** Most common bigrams of preposition and article and their orthographic chunks.

Standard bigram	Frequency	% chunked	% of different orthographic chunks
in der	349	51	ide/ida/idä (73 %), ir (27 %)
mit dem	333	45	mitem/metem/mitam (64 %),
			mitm/midm (32 %), mim (4 %)
auf der	246	12	ufe/ofe/ufä (57 %), ufde (40 %)
auf dem	242	57	ufem/ofem/ufam/ufum (68 %),
			ufm/ofm (32 %)
auf die	221	11	ufd/uft (60 %), ufdi (40 %)
wegen dem	185	64	wegem/wegäm/wägäm (81 %),
			wegm/wägm (19 %)
mit der	183	14	mite (80 %), mitde (1.4 %)
in die	182	81	id/it/et (99 %)
für die	176	22	fürd/förd (99 %)
von der	144	22	vode/vodä (78 %), vor (31 %)
vor der	144	01	vorde (100 %)
an der	142	45	ade/adä (88 %)
bei der	136	32	bide/bidä (70 %), bir/ber (30 %)
an die	118	69	ad/at (95 %)

One could assume that this variation is due to phonotactics, and that some sound sequences resulting from appending an article to a preposition might be more or less preferred. After all, some prepositions are vowel-final, some consonant-final. Likewise, articles – regardless of definiteness in the case of gsw – can be consonant-initial or vowel-initial. Also, some attached articles form one or even two syllables (e.g., *in-ere* in-INDF:F:SG:DAT), whereas others are reduced to becoming part of the rhyme of the last syllable in the preposition (e.g., *uf-d* onto-DEF:F:SG:NOM). The importance of the syllable in language processing has been studied for a long time (e.g., Mehler et al. 1981; Shattuck-Hufnagel 2011), and phonological judgments about its structure seem to be linked to orthographic knowledge (Kolinsky et al. 2012). Thus, syllabification might play a role in visual word separation as well.

The high inconsistencies in the orthography, which are largely a consequence of dialectal differences, make it hard to account for phonological effects in all tokens. For instance, the bigram “an der” “at the” we find realized as *an dr*, *a dr*, *ar*, *a de*, *adä*, or *ade*, depending on dialect and writer preference. But these dialectal variants prime the actual written realizations, and thus potentially orthographic fusion. And in fact, the comparison of the orthographic chunks in Table 2 indicates that syllable structure have an influence on conjoint writing.

The prevailing number of written chunks displays CV syllable structure, whereas consonant clusters at the juncture seem to be disfavored. In particular, we find the preferred realizations of the bigrams with the highest chunking rate “in der,” “auf dem,” “wegen dem,” “in die,” and “an die” to be CV(CV), whereas their less favored variants include consonant clusters (*ufm/ofm* and *wegm/wägm*). Also, the very common bigram “vor der,” which involves a cluster at the juncture, is practically never written as a unit.

Prepositions are not only the largest word class that articles fuse with in the corpus but also the largest class preceding them in general. Another class commonly preceding articles, and which, therefore, constitutes potential hosts, are particles like *noch*, *ganz*, *so*, and *auch*. These four have each >100 occurrences followed by an article in the corpus, 771 in total. However, only very rarely do they form an orthographic unit with the article, we only find 53 of these combinations chunked (i.e., below 7 %). Among these, *so* “such” dominates the largest share, being orthographic host for 49 articles. To avoid hiatus in cases where the article starts in a vowel, speakers usually insert a nasal between vowels (Fleischer and Schmid 2006: 249), and this is also reflected in the orthography: *so* + ART is realized as *sone*, *sonen*, *sones*, *sonere*, or *somene*. Interestingly, this does not happen with noch, ususally *no* in gsw. The intricacies of epenthetic *n* in Alemannic has been described by Ortmann (1998: 58–65), who states that it is ungrammatical following certain lexemes, including *no*. This lexical restriction is mirrored in the orthography, a fact which underlines how orthographic chunking is substantially predefined by the phonological connection.

### 3.3 Articles and nouns

Common nouns in the singular, all definite nouns in the plural, as well as some proper nouns like personal names require an article in gsw. Due to the high frequency of article–noun combination, it is not surprising that they are among the most common orthographic chunks that deviate from the standard (cf. Table 1). In Section 2.2, we stated that according to previous literature, within a prepositional phrase we would expect articles to be more likely to fuse with the preposed preposition than with the postposed noun. So if one wants to look more closely into the article–noun combination, one should control for the preceding element, due to the potential attracting effect of a preposition.

And in fact, prepositions seem to have a magnetic effect on articles: out of all article–noun combinations that are preceded by something else than a preposition, 2966 in total, 259 are written as a unit (i.e., 9 %).15Again, instances of *demfall* and its variants, which also provide positive hits for this query, were excluded. This proportion is different from that of article–noun fusion within PPs: Table 3 displays different types of chunks within prepositional phrases. It shows that within PPs of the type preposition + article + noun (common or proper), we find all possible chunks, with their respective frequencies. In most cases, all three elements are written as separate units, but also very commonly, prepositions and articles are fused, as addressed in the previous section. And as expected, we find that this kind of chunking is by far more frequent than that of article onto noun, which makes up only around 1 % of PP realizations.

**Table 3:** Conjoint writing within prepositional phrases; colons indicate items being written together.

Chunking within PP	Frequency	Examples	Gloss
PREP ART N	1,956	us em huus, weg de Zit	out.of the house, because.of the time
PREP:ART N	1,487	wegem zmittag, ufde Pischte	because.of:the lunch, on:the slope
PREP ART:N	39	a dzit, för sRechtige	about the:time, for the:right.thing
PREP:ART:N	6	irStadt, idemfall	in:the:town, in:this:case

Looking into the data of chunked ART + NOUN combinations gives a more straight-forward picture than the one for prepositions and articles, in that there is less variation. With only very few exceptions,16To be precise, 10 exceptions, involving DEF.M.NOM (*drX* or *deX*), INDF.M (*eX, eiX*), and INDF.F (*eX, nX*). all articles chunked onto a postposed noun are either realizations of the article for the DEF.SG.F or DEF.PL, *die*, shortened to *d* or *t*, or of *das*, DEF.SG.N, shortened to *s* or *z*, irrespective of the initial letter of the noun. The phonetic realization of any of these variants is voiceless, as there is no voicing distinction in Swiss German obstruents, except between [f] and [v] (Reese 2007: 9). No other article has a shortened variant that cannot form a syllable nucleus. Thus, articles get chunked onto a following noun only if they don’t form a phonological syllable of their own.17Avoidance of a stand-alone letter can be excluded as a (sole) reason, as there are also free standing instances of the indefinite article, which are comprised of just one letter as well, namely *e*, *ä* or *n*.


The reverse is not true; however, the fact that an item is not a phonological syllable on its own does not necessarily lead to it being written in connection to an adjacent element. There are even more instances of shortened *die* and *das* as separate units than as parts of a written chunk: we find 274 instances of free-standing *s* or *z*, and 496 instances of *d* or *t*. There is also a notable number of cases where instead of a space, or in addition to a space, an apostrophe is used by some writers. According to Nübling (1992: 308–309), the use of an apostrophe means lower acceptance of the fusion of two items and signals a conscious violation of the norm.

So again, the chunking of articles and nouns is clearly associated to phonology, more precisely syllable structure, as the former are much more likely to be chunked onto the latter if they don’t form a syllable on their own. This is in contrast to what we found for the fusion of prepositions and articles: although a lot of the variation can be attributed to inference from phonotactics, it is not crucial if the article forms a syllable on its own or not.

At this is point, it is worth to briefly talk about other items preceding nouns and fusing with them. Table 1 shows the numbers and an example for preposed prepositions and adjectives, respectively. The relatively high number of chunks made up of prepositions and common nouns (PREP NN) is largely due to frequent idioms involving such a combination, such as *zobig* (to:evening, some variants are *zabig, zobi* or *zabe*), or *znacht* (to:night), both meaning either “in the evening” or “dinner.” There are also chunks of prepositions and proper nouns (PREP NE), which are almost exclusively examples like the one provided in the table, i.e., instances of *z* “at, in” combined with a place name.18The only two exceptions are *inTravers* “in Travers” and *bigoscht* “oh god,” lit. “close to god.” These 34 tokens make up 17 % of this particular preposition + noun combination in the whole corpus.

Conjointly written adjectives and nouns are to a large part very common, and more or less lexicalized combinations as well: most of them are variants of collocations like *andrsmal*, *letschmal* or *negschmal* (another:time, last:time, and next:time), *guetemorge* (good:morning) or *guetnacht* (good:night), or *schönabe* (nice:evening, meaning “have a nice evening”). The remaining few chunks of this kind can be explained by analogy to unit prefixes (e.g., *gratisruusch* free.of.charge:high), or cases where one could actually argue whether the first part is actually an adjective, or rather a noun, rendering a perfectly normal German noun–noun compound (e.g., *scheisskolleg* or *scheissarbet*, shit:friend and shit:work, respectively).

### 3.4 Conjunctions and ponouns

The following two sections deal with written chunks one part of which is a (reduced) pronoun. These pronominal forms are not confined to a particular case (NOM, ACC, or DAT) and can refer to different grammatical roles. This is because word order is very flexible, and as in many Germanic languages, pronouns are rarely dropped. Also, in Section 3.5 below, we will encounter verb forms that entail a bound subject form, additionally to showing agreement in the verb ending.

But before looking into the biggest group of nonstandard chunks from Table 1, verbs + pronominals, we will deal with another type of part of speech that the latter often fuse with, namely conjunctions. Some findings for this group will also be relevant for the next section. Nübling (1992: 14) states that conjunctions in Allemanic can bind all pronominals to them, and also several at a time. As an example she provides *wil = er = em = s gseit het* “because he told him so” with one chunk consisting of the conjunction and three pronominal forms: because = he = him = it.19The order of elements is only partly fixed in this chunk: whereas the nominative *er* has to follow the conjunction immediately, the other two arguments could also swap: *wil = er = s = em*. Such a case occurs in the corpus only once, shown in (5).

(5)

**Table:** 

I	ha	nüm	gwüßt	öbisdr	scho	am	tel	verzellt	ha
1SG	AUX	NEG	know.PTCP	if:I:it:you	already	on	phone	tell.PTCP	AUX
“I didn’t remember if I had already told you about it on the phone” [6687]									

Irrespective of orthographic fusion, a conjunction followed by three pronominals is quite rare, and there are only six occurrences in the whole corpus. Interestingly, none of them are four orthographic words, but (besides the one just described) either two or three, so there is always some splicing with this combination. This is due to the fact that also two pronominals can be combined, but seemingly only under one condition: similar to what we found for the short form *z/s* for the article *das* in Section 3.3, all instances of two conjointly written pronominals without lexical head (such as *ers, drs, ichs*, with a total 88 occurrences in the corpus, PPER PPER in Table 1) have *s*, short for *es* (3SG.N ACC or NOM), as the second element.

A 3SG pronoun is overall very common in the corpus,20The top ten pronominals in the corpus in descending order: *ich* 1SG.NOM, *es* 3SG.N.NOM/ACC, *du* 2SG.NOM, *dir* 2SG.DAT, *wir* 1PL.NOM, *dich* 2SG.ACC, *mir* 1SG.DAT, *mich* 1SG.ACC, *sie* 3PL.NOM/ACC or 3SG.F.NOM/ACC, *uns* 1PL.ACC/DAT not least because it is also the form of expletive subjects in phrases containing existentials, weather expressions or the very common greeting corresponding to English “How are you” (*wie gahts/wie gehts* in gsw). It is also the second item in the most common orthographic units of a conjunction and a pronominal, namely standard *wenn es*, *wie es*, and *weil es*, with >90 % chunking rate each. This is shown in Table 4, which lists all bigrams of conjunction + pronominal with more than ten occurrences in the corpus, and the proportion of conjointly written ones.

**Table 4:** Chunking of conjunctions and pronominals.

Bigram	Frequency	Chunked in %	Examples orthographic chunks
dass ich	323	53.6	dassi, dasi
wenn du	291	57.4	wend, went, wennd, wännt
wenn ich	196	58.7	wäni, weni, wenni, wänni
dass du	182	27	dassd, dasd, dasd’, dast, daßd
wenn es	132	97	wenns, wens, wes
dass es	99	48.5	dasses, dases
ob ich	82	52.4	obi, öbi
wie es	81	90.1	wies, wis, wiäs
weil ich	77	44.2	wili, weli, willi, welli
dass wir	70	17.1	dasmer, dassmer, daßmer
wenn wir	57	50.9	wämer, wämmer, wemmer
wie ich	35	25.7	wieni, wini
ob du	34	35.3	obd, öbd
wenn ihr	33	33.3	wenner, wänner
ob es	31	96.8	obs, öbs
dass er	29	31	dasser, daser
falls du	28	57.1	fallsd, falsd, falst
dass sie	28	3.6	daßi
wenn sie	22	9.1	wenns, wäns
weil es	21	90.5	wils, wills, wells
falls es	19	31.6	fauses, fallses, falses
ob sie	19	31.6	obsi, obs, öbs
falls ich	19	21.1	fallsi, falsi
ob wir	18	16.7	obmer, öbmer
wenn er	17	23.5	wenr, wenner
da ich	17	0	
weil du	15	40	wilt, weld
damit ich	15	20	damiti
obwohl ich	14	35.7	obwohli
weil er	14	21.4	weler, wilr, weller
falls ihr	13	23.1	fallser
ob er	13	15.4	öber, obr
weil sie	13	7.7	wils
sobald ich	12	25	sobaldi
dass dich	12	8.3	

On the other hand, *dass es* and *falls es*, which are also a very frequent combinations, include the 3SG.N as well, but because the conjunctions end in an alveolar fricative, the vowel is realized, the pronominal forms its own syllable, and this is probably why the space is omitted in roughly half of the cases only.

Like 3SG.N *es/ös*, 3PL *sie* can be realized as *s* (e.g., in *Aber sus sind* ***s*** *fein* [11964] “But apart from that, **they** are tasty.”), and all instances of this reduced form are fused with the preceding conjunction (i.e., there is no free-standing *s* or *‘s* for 3PL after conjunctions). This is different for *es*, which occasionally occurs as a free standing reduced form in the corpus. Thus, there seems so be a lexically conditioned difference between the phonologically identical forms.

The 2SG.NOM behaves similar to the 3SG: all fused forms are realized as *d* or *t*. However, not all separately written ones have the full form *du*, but there are a few instances of *d* or *‘d* preceded by a space. Yet again, nonsyllabicity seems to be the main criterion for chunking. In contrast to the nominative form, the accusative *dich* cannot be reduced to the initial plosive but renders *di(i)* in the short form. In the few instances where it follows a conjunction in the corpus, it is not fused with the preceding item.

The examples from the corpus in Table 4 also illustrate that hiatus is in some cases avoided at the conjunction of two items in writing, just like what we have seen for vowel-final prepositions and vowel-initial articles in Section 3.2. Between a final vowel in a conjunction and 1SG *i*, some speakers insert an epenthetic *n*, and these cases are orthographic units (*wieni/wini*). Variants without *n*, on the other hand, are realized with space insertion, the form *wi ni* occurs only once in the corpus. Interestingly, this does not happen after the other vowel-final conjunction *da* “because”; after consulting with some native speakers, this conjunction seems to be rather rare is gsw overall, and its use in the corpus might be strongly influenced by Standard German. The other pronominals that start in a vowel (such as *er/är* 3SG.M or *er/ihr* 2PL) are too rare in combination with *wie* to appear in the table, but the epenthetic *n* can also be found there, rendering the forms *wiener/wianer* in contrast to *wie er*.

There are other byproducts of phonological processes observable in the data. For instance, we find assimilation at the juncture in the orthographic chunks of the standard bigram *wenn wir* in Table 4. The 1PL.NOM is usually either *mir* or *mer* in gsw. The final nasal of the conjunction is assimilated to the first nasal of the pronominal. Crucially, with only one exception (*wännmer*), all instances of this bigram in its chunked form entail this assimilation, rendering tokens like the one in the table. The reverse is also true: none of the variants separated by a space have the assimilated nasal; all have an orthographic < n>. The phonological process of assimilation might make it hard to actually separate the individual items here and thus work in favor of orthographic fusion: Assimilation is one of a number of phonological processes, which can weaken and thus blur morpheme boundaries (Kabak 2014) and make it hard to parse the individual parts of a chunk. So for, for speakers of gsw, it is potentially hard to dissect a chunk like *wemmer*.

As was shown in Table 1, bound pronominals also occasionally fuse with other parts of speech, namely wh-words and prepositions. For the first group, the same as we stated for conjunctions seems to hold true: the most frequent chunks are *woni* (*wo*+1SG) and *wod* (*wo*+2SG). As for prepositions and pronominals, this group is very diverse, with too many types of bigrams with too few tokes each to make any generalizations.

### 3.5 Verbs and pronouns

As can be concluded from Table 1, verbs (full, auxiliary, and modal taken together) followed by pronominals have by far the highest rate of nonstandard orthographic fusion. Looking into the top ten most frequent bigrams in Table 5, it becomes clear that it is not frequency of adjacent occurrence alone that is accountable: even with highly frequent bigrams like *wünsche dir* “wish to you,” *freue mich* “am happy,” or *liebe dich* “love you,” the proportion of chunked tokens is vanishingly small.

**Table 5:** Ten most frequent combinations of verbs and postposed pronominals.

Standard bigram	Frequency	% chunked	Examples orthographic chunks
geht es	729	96.8	gahts, gohts
wünsche dir	479	4.4	wünschder, wünschdr
habe ich	464	79.5	hani
freue mich	370	7.6	freumi
liebe dich	314	<0	liebdi
bin ich	266	71.4	bini
habe es	201	96.5	has, hans
kann ich	168	66.0	chani
ist es	166	70.5	isches, ischs
haben wir	145	85.5	hämmer, hämr

Syllabicity of the pronominal seems again one of the drivers for chunking: the pronominal for 3SG.N *es* (realized as *s* mostly, compare Section 3.4), leads to the highest scores, and is frequently chunked even after another coronal fricative in the fused form *ischs*. There is also a large proportion of chunks involving the 1SG *i* or *y*. In forms like *hani* 21The nasal in this belongs to the stem of the verb for “to have,” i.e., it is not epenthetic in this case. and *chani*, the verb-final consonants are resyllabified and become the onset for the nucleus allocated by the vowel of the pronominal.

The pronominal for the 1PL.NOM is very frequently chunked to a preceding verb, not only in the example *haben wir* provided in the table: of 1067 bigrams involving a finite verb and *wir* in the corpus, 726 are written as a unit. This can be partly explained by assimilation of the last segment of the verb, similar to what we have shown for conjunctions and pronominals in the preceding section. The coda of the last syllable of the verb often assimilates to the onset nasal of *mr* or *mer*, rendering *chömer* from *chönd* “can.1PL,” *machemer* from *mached* or *machend* “do.1PL,” *gömmr* from *gönd* “go.1PL,” or *wämmer* from *wänd* “want.1PL.” Assimilation is an overall very common feature of gsw, as compared to Standard German (Reese 2007: 12). Again, assimilation probably makes it harder to parse the individual parts.

As mentioned above, pronominals referencing any grammatical role can be reduced and orthographically fused. This leads to the formation of chunks, which, from a morphological point of view, express the categories of person and number of the subject twice. For instance, *bini* has to be correctly glossed as be.1SG:1SG. This orthographic fusion illustrates that gsw allows for complete morphosyntactic identity of adjacent elements within a prosodic unit, something which is prohibited in a number of other languages, including the Germanic language Dutch. There, agreement in the verb is suspended in a VS context with pronominal subjects, as a kind of haplology-effect (Nevins 2012: 92–94). As for gsw, writers don’t even hesitate to also visually express this kind of semantic doubling within a single unit.

As gsw is a verb-second language, pronouns (mainly, but not only, those referencing the subject) can also precede the verb in declarative main clauses. The following section will go into the differences in fusion of preposed versus postposed functional elements.

### 3.6 Nouns versus verbs and their aptitude for suffixes

The previous sections have dealt with the most frequent types and tokens of written chunks in gsw that deviate from the Standard German written standard. Most of the types of chunks dealt with so far, i.e., the most common ones displayed in Table 1 in Section 3.1, involve phonologically reduced forms of function words, pronominals and articles. The table and the subsequent analysis have not only pointed to these particular parts of speech often not being realized as independent orthographic words but also that they most commonly attach to a preceding lexical item or preposition.

In Section 1, the question has been raised whether the behavior of these elements might be a reflex of the cross-linguistic preference for suffixation over prefixation. More precisely, we asked whether we find more fusion of reduced forms with their preposed element as compared to a postposed element, as well as whether we find differences between verbs and nouns as lexical hosts.

At first glance, the data speak in favor of the latter. With nouns, we found that the items that most often attach to them are preposed articles, with verbs, on the other hand, it is postposed pronominals. However, in prepositional phrases, where there is an actual choice for articles to attach to either a preceding or to a following host, they rather fuse with the preposition than with the following noun, which would speak in favor of the general suffixing preference (cf. Section 3.2).

In order to clarify whether it is in fact the noun that triggers anterior attachment, and not the article which prefers to be an appendage, one would need to compare instances of the same group of functional elements that can both either precede or follow the same group of heads. But in gsw, there aren’t many postposed function words that have a noun as a lexical head: postpositions are rare (and rarely used), and practically nonexisting in the corpus.

The only cases where one can look into suffixing versus prefixing (in an orthographic sense) with conjoint elements of the same type are verbs and pronouns, as they can occur in either order respective to each other. Examples for orthographically suffixed pronouns are provided in Table 5; for the reverse order, there are the examples *sgitt* and *sisch* in Table 1, but other pronouns apart from *s* behave the same, such as in *chchum* or *ichum*, both being 1SG:come.1SG.

In Table 6, we compare the numbers for chunked and nonchunked tokens of this combination. It becomes immediately evident that there is a relationship between the order of verb and pronominal on the one hand and space insertion on the other hand. This relationship is also statistically significant: the chi-square statistic is 4,151.382, with a p-value <0.00001, significant at p < 0.05.

**Table 6:** Pre- versus postposed pronominals and verbs.

	Verb pro	Pro verb
Chunked	4,869	58
Separate	6,545	7,502
Total	11,414	7,560

However, we cannot assume from these numbers that this relationship is an immediate one, i.e., that the inclination toward postposed chunking is triggered by the verb itself. First of all, not all reduced forms of pronominals of gsw can be used before a verb, for instance, the reduced form of the 2SG.NOM *d/t* never occurs clause initially. If one does find this form before a verb, there is usually a conjunction in the first position of the clause to which *d/t* attaches. Also, all prechunked pronominals are *i* 1SG.NOM or *s* 3SG.N. In the case of the latter, one could again argue that syllable structure is the cause here, with *s* forming the first part of the onset of the following syllable.

Himmelmann (2014) has argued that the asymmetry generally lies in prosodic phrasing. The presence of prosodic boundaries after a function word preceding a functional head can disrupt a potential fusion process. However, prosodic boundaries rarely occur after a lexical element and the reduced function word, allowing them to seamlessly fuse. So assuming that conjoint spelling mirrors prosodic phrasing, the difference is probably not inherent to any parts of speech.

## 4 Discussion and conclusions

The notion of the “word” has been acknowledged to be a complex one and has been debated by linguists, especially typologists, for quite some time now. In many contexts, the word is equated with an orthographical unit, meaning that it is defined by its written form. This is particularly common in fields like natural language processing (NLP) and psycholinguistics. Also language description and typology have been influenced by orthographic conventions (e.g., noted by Gil 2020; Haspelmath 2023). However, this narrow definition of a word can overlook the many other aspects of language that are not represented in writing, such as phonological processes and lexical restrictions thereon.

Our investigations of a corpus of text messages have revealed that writers of a nonstandard variety of German orient a lot toward phonology when deciding to write certain elements conjointly. We found that processes like assimilation and epenthesis promote orthographic fusion, potentially because they make the dissection of the individual parts of speech more difficult. Also, some common combinations are considered as units in gsw, similar to compounds and particle verbs, which would be written conjointly according to the rules of the standard. Frequency seems to play only a minor part. Our observations have been in line with the hypotheses that firstly, chunking is overall more frequent in written gsw than in the written standard (i.e., there are fewer orthographic words) and secondly, that the parts of speech this mostly pertains to are those that have been analyzed as clitics in the previous literature, and the status of which has been described as being in transition from syntax to morphology (Nübling 1992).

Our observation that Alemannic dialect writers tend to prioritize prosody and phonology over syntax when deviating from standard German space insertion is in line with findings dealing with space insertion in old writings and inscriptions. Busch and Fleischer (2015) looked into different writings from varieties of Old High German and Old Low German and found, among other things, that there are often no spaces after prepositions, and that there are lexically and phonologcially conditioned differences between different prepositions. Also, Crellin (2022) shows for ancient Northwest Semitic writing systems and Epigraphic Greek how the written units largely correspond to different targets on a prosodic level rather than on a syntactic one.

These are valuable insights, which have important implications for word-token based tasks, as well as for typological research, which is centered around words as units. Relying more heavily on prosody and phonology, Alemannic writing challenges the validity of orthographic units as proxies for syntactic building blocks in language. This has consequences, for instance, for measuring internal complexity, word boundary detection, or self-paced reading tasks.

Firstly, in the domain of NLP, where systems predominantly equate orthographic words with basic building blocks, our findings highlight that caution is in order. The reliance on orthographic information to segment text into words and phrases may inadvertently overlook the profound influence of phonology in shaping these processes. Integrating phonological information into NLP models becomes imperative, not only for enhancing performance but also for unraveling the nuanced variations in hierarchical structures within the same language system, contingent upon differences in space insertion. This nuanced understanding serves as a warning against the oversimplification of language structures when solely relying on orthographic representations.

Secondly, the dynamic nature of word boundaries in Alemannic dialectal writing suggests that readers cannot solely depend on orthographic cues for identifying word boundaries. This variability may impact reading patterns in experiments like self-paced reading tasks. While we observed robust tendencies for certain elements to be chunked together as orthographic words, it is crucial to note that no single phonological, lexical, or pragmatic parameter consistently predicts space insertion. The observed variation, reflective of the inherent complexity in people’s cognitive processing, remains unaccounted for in traditional orthographic rules. This discrepancy poses a potential challenge, as relying solely on orthographic representations may fail to capture the intricacies of language processing in the human brain.

This, however, would in turn presuppose that phonology and prosody play an equally important role in reading (i.e., perception) as in writing (i.e., production). Research has shown that the parts of our brain involved in processing spoken language and the parts involved in processing written language are closely connected. This connection increases with better reading skills (Azaiez et al. 2022). What is more, literacy (in the sense of the individual’s ability to read and write) actually changes our brains. There is no brain region that has evolved specifically for reading or writing, but other areas with similar functions (e.g., those responsible for meaning as well as those responsible for articulation) have been neuronally recycled in order to take on these tasks (Dehaene 2005; Dehaene et al. 2010). And it is probably fair to assume that the acquisition of writing plays some part in forming these connections involved in the processing of the written word to arise.

## Abbreviations

Abbreviations generally follow the Leipzig Glossing Roles, all further abbreviations are the following:

PREP: preposition
CONJ: conjunction
MOD: modal particle
NE: proper noun
NN: common noun
PPER: personal pronoun
PTCP: participle
NEG: negative particle
PTZU: particle “zu”
VAFIN: finite auxiliary
VMFIN: finite modal verb
VVFIN: finite full verb
WH: question words & relative pronouns
